# Risk Factors of Non-en Bloc Resection and Non-R0 Resection During Endoscopic Resection in the Treatment of Superficial Duodenal Epithelial Lesions

**DOI:** 10.3389/fonc.2022.881815

**Published:** 2022-05-20

**Authors:** Shifeng Fu, Jian Gong, Mei Zhou, Yongjun Wang, Deliang Liu, Yuyong Tan

**Affiliations:** ^1^ Department of Gastroenterology, the Second Xiangya Hospital of Central South University, Changsha, China; ^2^ Research Center of Digestive Disease, Central South University, Changsha, China

**Keywords:** superficial duodenal epithelial lesions, endoscopic resection, En bloc resection, R0 resection, risk factor

## Abstract

**Background:**

Superficial duodenal epithelial lesions are precancerous lesions of duodenal carcinoma. Upper gastrointestinal endoscopy has been widely used in the screening and treatment of this disease. This article will collect the data of patients who underwent endoscopic resection of superficial duodenal epithelial lesions in our hospital from 2010 to 2021, aiming to describe the efficacy and safety of endoscopic resection, as well as to explore the risk factors of non-en bloc resection and non-R0 resection.

**Methods:**

Patients who underwent endoscopic resection for superficial duodenal epithelial lesions in our hospital from January 2010 to September 2021 were selected. The curative effect was expressed by the en bloc resection rate and R0 resection rate. The safety was expressed by intra- or postoperative complications, such as bleeding and perforation. The potential risk factors of curative effect were analyzed by logistic regression.

**Results:**

A total of 137 patients were included. The en bloc resection rate was 95.62% (131/137), R0 resection rate was 91.97% (126/137), the postoperative bleeding rate was 2.19% (3/137), and no postoperative perforation was found. The histology result of ectopic gastric mucosa was the risk factor of non-en bloc resection (OR: 8.86, 95% CI: 1.38-56.92); the lesion size ≥2 cm was the risk factor of non-R0 resection (OR: 12.55, 95% CI: 2.95-53.38).

**Conclusion:**

Endoscopic resection is a safe and effective method for the treatment of superficial duodenal epithelial lesions. The histology result of ectopic gastric mucosa was the risk factor of non-en bloc resection and the lesion size ≥2 cm was the risk factor of non-R0 resection.

## Introduction

Superficial duodenal epithelial lesions (SDELs) are rare and are considered as precancerous lesions of duodenal carcinoma (1, 2). Usually, patients with SDELs do not have specific symptoms and signs, so early-stage lesions are difficult to be found. However, in recent years, with the widespread application of upper gastrointestinal endoscopy in physical examinations and the development of new endoscopic techniques, the detection rate of SDELs has gradually increased (3). The endoscopic resection (ER) is also widely used in the treatment of SDELs, which is considered as a minimally invasive treatment that can achieve cure (4–6). ER is usually divided into two types, endoscopic mucosal resection (EMR) and endoscopic submucosal dissection (ESD). Compared with the former, the latter can remove deeper lesions, but also has a higher incidence of postoperative complications, such as bleeding and perforation (7). This article will report on the efficacy and safety of SDELs patients treated by ER in our hospital from January 2010 to September 2021, as well as to explore potential risk factors of complications and treatment effects.

## Materials and Methods

### Research Design and Population

This study is a retrospective analysis, selecting patients with SDELs who underwent ER in our hospital (a high-volume institution that actively carries out endoscopic treatment) from January 2010 to September 2021. The selection criteria include: 1) duodenal lesions were found by upper gastrointestinal endoscopy; 2) upper gastrointestinal endoscopy, endoscopic ultrasonography or computed tomography confirmed that the lesions were limited to the mucosal layer or superficial submucosa layer; 3) patient signed and agreed for ER. Exclusion criteria include: 1) patients with incomplete information; 2) patients with contraindications of ER, such as coagulation dysfunction, cardiopulmonary insufficiency, etc; 3) patients with multiple lesions in the duodenum. The collected data include: basic information of the patient (gender, age, smoking, etc.), symptoms, characteristics of duodenal lesions (location, size, etc.), surgery and treatment situations (en bloc resection, R0 resection, positive margins, pathology results), postoperative complications (postoperative bleeding and postoperative perforation), and the patient’s background diseases. All patients have been fully informed and signed informed consent. This study was approved by the ethics review committee of the Second Xiangya Hospital of Central South University.

### Definition

En bloc resection is defined as the lesion is completely removed, and there is no segmentation or partial resection. R0 resection is defined as: on the basis of complete resection of the lesion, no cancerous tissue and components are found on the horizontal and vertical edges. Postoperative bleeding is defined as: patient has hematemesis, melena, and unexplained hemoglobin drop more than 2g/dL after ER (8). Postoperative perforation is defined as: the patient had sudden abdominal pain after resection of the lesion, accompanied by retroperitoneal pneumatosis or free gas detected on abdominal computed tomography scan or radiographs (8, 9). Polyps are defined as: discrete abnormal tissue masses that protrude into the lumen of the digestive tract, and duodenal polyps include neoplastic and non-neoplastic lesions. The polyps mentioned in the histological results in this study refer to non-neoplastic polypoid lesions (10).

### ER Procedure

We choose specific ER method according to the size, location of the lesion and the results of biopsy. ESD is usually recommended for lesions larger than 2cm (11). If the results of biopsy tend to be non-neoplastic lesions or LGIN, EMR will be selected, otherwise ESD will be selected. For lesions larger than 2 cm and had technical difficulty to perform ESD, EPMR will be selected.

**Figure 1:**
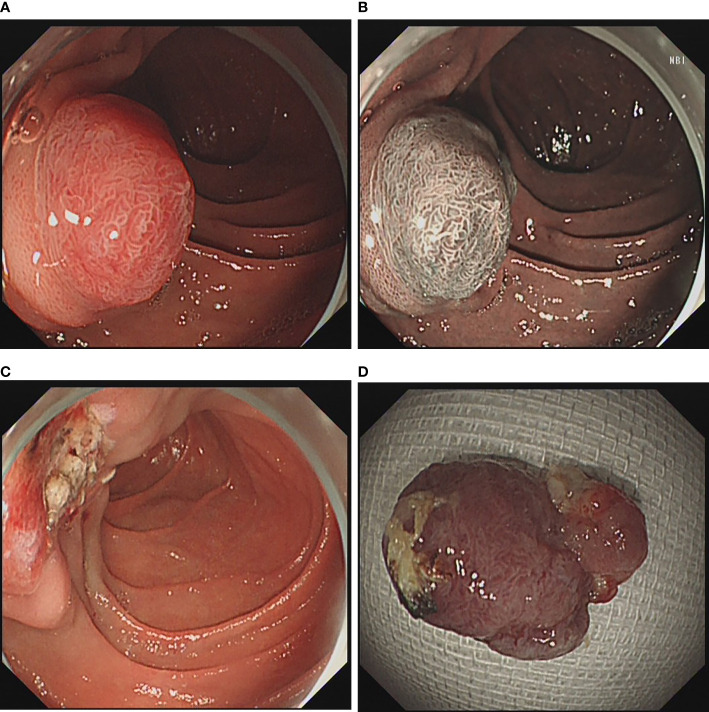
EMR for duodenal papillary lesions. **(A)** Lesion at the duodenal papillary; **(B)** Duodenal papillary lesion in NBI mode; **(C)** Wound after lesion resection; **(D)** Lesion after resection.

**Figure 2:**
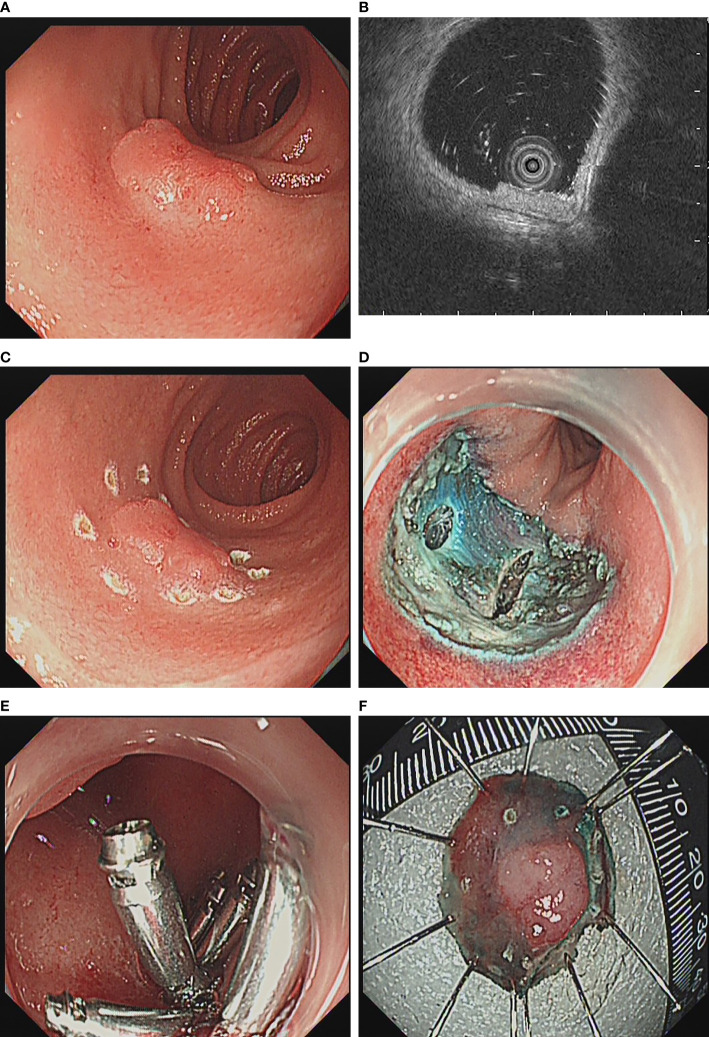
ESD for duodenal bulb lesions. **(A)** Lesion at the duodenal bulb; **(B)** Endoscopic ultrasonography revealed that the lesion was confined to the mucosal layer; **(C)** Dual knife was used to mark around the lesion; **(D)** Wound after lesion resection; **(E)** Close the wound with a metal clip; **(F)** Lesion after resection.

The instruments used for ER include single channel endoscope (GIFQ260J, GIF-H290; Olympus) with a transparent cap (D201-11802; Olympus), high-frequency generator (ICC 200, ICC 300, or VIO 200D; ERBE), argon plasma coagulation device (APC300; ERBE; Tübingen, Germany), carbon dioxide inflator (UCR; Olympus), thermal biopsy forceps (FD-1U-1; Olympus), injection needle (NM-4L-1; Olympus), snare (SD-221L-25, SD-230–20; Olympus) dual knife (KD650Q; Olympus), insulation-tip knife (KD-611L; Olympus), hybrid knife (ERBE; Tübingen, Germany) and hemostatic clips (HX-600-90, HX-600-135; Olympus). During EMR, 100 ml of normal saline, 1 ml of indigo rouge and 1 ml of adrenaline solution were injected into the submucosal layer, and then resect the lesion with a snare. (**Figure 1**) If it was piecemeal resected the lesion with a snare, it was endoscopic piecemeal mucosal resection (EPMR). For ESD, dual/hybrid knife was used to mark around the lesion, followed by administration of a submucosal injection. After precutting the mucosa and submucosa, dual knife, hybrid knife or insulation-tip knife was used to dissect the lesion. Then, if the muscularis propria was unintentionally damaged or there was a risk of bleeding and perforation, the mucosal defect was closed with metal clip or metal clip together with endoloop. The resected lesions were embedded in paraffin and sectioned for pathological assessment (**Figure 2**).

### Statistical Analysis

The data in this article will be statistically analyzed through SPSS 18.0. Among them, categorical variables will be expressed by frequency, and continuous variables will be expressed by mean ± standard deviation. For the screening of risk factors, first carry out univariate logistics regression analysis, and then incorporate statistically significant indicators into multivariate logistic regression. P<0.05 is considered statistically significant.

## Results

### Basic Information of Cases

**Figure 3:**
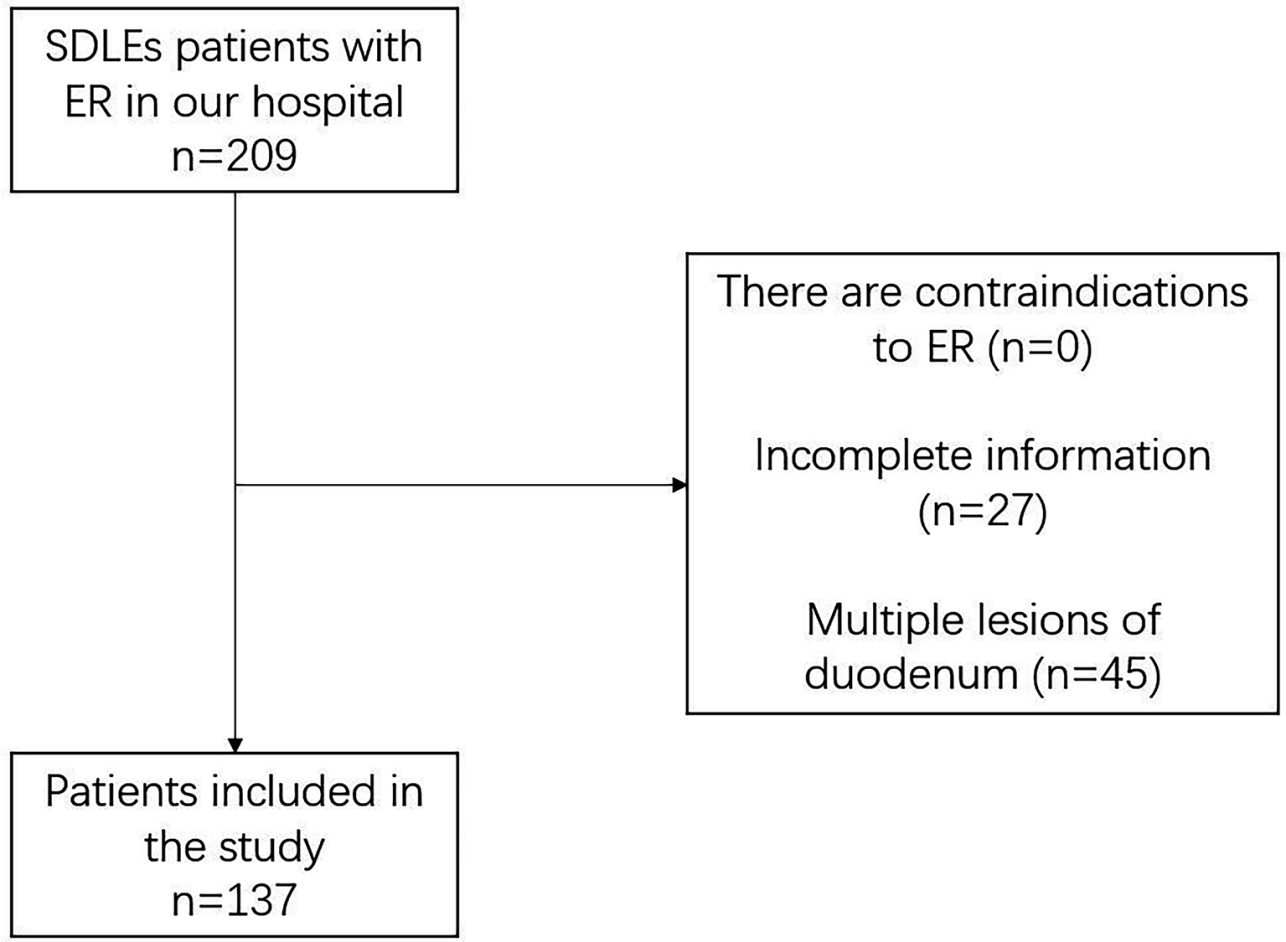
Patient screening process chart.

A total of 137 patients in our hospital from January 2010 to September 2021 were collected (**Figure 3**), including 84 males and 53 females, with an average age of 52.92 ± 12.90 years, including 114 who underwent EMR, 19 who underwent ESD, and 4 who underwent EPMR. The main symptoms were abdominal pain (42.33%, 58/137), physical examination findings (27.00%, 37/137), and abdominal distension (21.90%, 30/137). 41 patients (29.93%) had concomitant diseases, of which hypertension (19.71%, 27/137) was the most common. A total of 36 people smoked (26.28%). (**Table 1**). 54 patients (39.42%) completed preoperative biopsy, including 1 case (1.85%) of lymphoma, 5 cases (9.26%) of Low-grade intraepithelial neoplasia (LGIN), 7 cases (12.96%) of high-grade intraepithelial neoplasia (HGIN), 10 cases (18.52%) of polyps, 7 cases (12.96%) of inflammation, 4 cases (7.41%) of ectopic gastric mucosa and 20 cases (37.04%) of adenomas, of which 9 cases were complicated with LGIN and 3 cases were complicated with HGIN.

**Table 1:** Characteristics of the 137 cases of SDELs treated with endoscopic resetion.

	EMR (n=114)	ESD (n=19)	EPMR (n=4)	Total (n=137)	P
Gender (%)					<0.001
Male	71 (62.28)	9 (47.37)	4 (100.00)	84 (61.31)	
Female	43 (37.72)	10 (52.63)	0	53 (38.69)	
Age (years)	53.17 ± 12.31	53.35 ± 12.15	42.50 ± 27.60	52.92 ± 12.90	0.262
Lesion Diameter (cm)	1.11 ± 0.60	2.49 ± 1.47	3.18 ± 0.24	1.35 ± 0.95	<0.001
Lesion Location (%)					<0.001
Bulb	62 (54.39)	7 (36.84)	4 (100.00)	73 (53.28)	
Descending Portion	28 (24.56)	11 (57.89)	0	39 (28.47)	
Junction Of The First And Second Portions	23 (20.18)	1 (5.26)	0	24 (17.52)	
Horizontal Section	1 (0.88)	0	0	1 (0.73)	
Histology (%)					<0.001
LGIN	8 (7.02)	2 (10.53)	0	10 (7.30)	
HGIN	0	2 (10.53)	0	2 (1.46)	
Adenoma	24 (21.05)	4 (21.05)	0	28 (20.44)	
Polyp	53 (46.49)	3 (15.79)	1 (25.00)	57 (41.61)	
Ectopic Gastric Mucosa	3 (2.63)	2 (10.53)	2 (50.00)	7 (5.11)	
LGIN with Adenoma	23 (20.18)	4 (21.05)	1 (25.00)	28 (20.44)	
HGIN with Adenoma	1 (0.88)	1 (5.26)	0	2 (1.46)	
HGIN with Adenocarcinoma	0	1 (5.26)	0	1 (0.73)	
Polyp with Ectopic Gastric Mucosa	2 (1.75)	0	0	2 (1.46)	
Vienna Classification (%)					<0.001
Category 1	59 (51.75)	5 (26.32)	2 (50.00)	66 (48.18)	
Category 3	55 (48.25)	10 (52.63)	1 (25.00)	66 (48.18)	
Category 4.1	0	3 (15.79)	1 (25.00)	4 (2.92)	
Category 4.2	0	1 (5.26)	0	1 (0.73)	
En bloc Resection (%)	112 (98.25)	19 (100.00)	0	131 (95.62)	<0.001
R0 resection (%)	110 (96.49)	16 (84.21)	0	126 (91.97)	<0.001
Positive Margin (%)	2 (1.75)	3 (15.79)	0	5 (3.65)	<0.001
Complications (%)					
Intraoperative Perforation	0	0	0	0	
Postoperative Bleeding	3 (2.63)	0	0	3 (2.19)	
Postoperative Perforation	0	0	0	0	
Clip After ER (%)	56 (49.12)	15 (78.95)	1 (25.00)	72 (52.55)	<0.001
Symptom (%)					<0.001
Abdominal Pain	47 (41.23)	10 (52.63)	1 (25.00)	58 (42.33)	
Abdominal Distention	27 (23.68)	2 (10.53)	1 (25.00)	30 (21.90)	
Abdominal Discomfort	4 (3.51)	1 (5.26)	1 (25.00)	6 (4.38)	
Belch	4 (3.51)	0	0	4 (2.91)	
Acid Reflux	2 (1.75)	1 (5.26)	0	3 (2.19)	
Heartburn	2 (1.75)	0	0	2 (1.46)	
Vomit	1 (0.88)	0	0	1 (0.73)	
Black Stool	2 (1.75)	1 (5.26)	0	3 (2.19)	
Hiccup	1 (0.88)	0	0	1 (0.73)	
Nausea	1 (0.88)	0	0	1 (0.73)	
Physical examination found	30 (26.32)	6 (31.58)	1 (25.00)	37 (27.00)	
Background Diseases (%)					<0.001
Hypertension	21 (18.42)	6 (31.58)	0	27 (19.71)	
Diabetes	10 (8.77)	1 (5.26)	0	11 (8.03)	
Coronary Heart Disease	4 (3.51)	0	0	4 (2.92)	
Cerebrovascular Diseases	1 (0.88)	0	0	1 (0.73)	
Hepatitis B	3 (2.63)	0	0	3 (2.19)	
Cigarette (%)	28 (24.56)	6 (31.58)	2 (50.00)	36 (26.28)	<0.001

EMR, endoscopic mucosal resection; ESD, endoscopic submucosal dissection; EPMR, endoscopic piecemeal mucosal resection; LGIN, low-grade intraepithelial neoplasia; HGIN, high-grade intraepithelial neoplasia.

### Efficacy and Safety

73 cases (53.28%) were located at the bulb, 39 cases (28.47%) were located at the descending portion, 24 cases (17.52%) were located at the junction of first and second portions, and 1 case (0.73%) were located at the horizontal section. The histology results showed that there were 59 cases (43.07%) of polyps, including 2 cases (1.46%) with ectopic gastric mucosa. There were 58 cases (42.34%) of adenoma, including 28 cases (20.44%) with LGIN and 2 cases (1.46%) with HGIN. There was 1 case (0.73%) of adenocarcinoma with HGIN. There were 10 cases (7.30%) of simple LGIN, 2 cases (1.46%) of simple HGIN and 7 cases (5.11%) of simple ectopic gastric mucosa. Among the patients who completed the biopsy, 40 cases (74.07%) had consistent postoperative histological results with the biopsy results, while the remaining 14 cases had inconsistent pre- and postoperative histological results. Among the 14 patients with inconsistent histological results, 7 cases (12.96%) had lighter histological results than the biopsy results and 7 cases (12.96%) had more serious histological results than the biopsy results. 131 cases (95.62%) underwent en bloc resection, 126 cases (91.97%) underwent R0 resection, and 5 cases (3.65%) had positive margin. Postoperative bleeding was 3 cases (2.19%) and no intraoperative or postoperative perforation to be found. A total of 72 cases (52.55%) used metal clips after ER (**Table 1**).

### Risk Factors of Complications

Univariate logistic regression analysis was conducted on the risk factors of potential surgical complications. The results are shown in **Table 2**. No statistically significant risk factors were found in the patients included in this research.

**Table 2:** Univariate logistic regression of postoperative complications.

Postoperative Bleeding	OR	95%CI	P
Gender (Female)	0.788	0.070-8.916	0.848
Age (≥60)	–	–	0.998
Lesion Location (Bulb)	–	–	0.997
Lesion Location (Descending Portion)	5.243	0.462-59.566	0.181
Lesion Location (Junction Of The First And Second Portions)	2.413	0.210-27.743	0.480
Lesion Location (Horizontal Section)	–	–	1.000
Lesion Size (≥2cm)	–	–	0.998
Histology (LGIN)	–	–	0.998
Histology (HGIN)	–	–	0.999
Histology (Adenoma)	2.786	0.247-31.481	0.408
Histology (Adenocarcinoma)	–	–	1.000
Histology (Polyp)	0.840	0.074-9.502	0.888
Histology (Ectopic Gastric Mucosa)	–	–	0.999
En bloc Resection	–	–	0.999
R0 resection	–	–	0.999
Positive Margin	–	–	0.999
Hypertension	–	–	0.998
Coronary Heart Disease	–	–	0.999
Diabetes	–	–	1.000
Cerebrovascular Diseases	–	–	0.999
Hepatitis B	–	–	0.999
Cigarette (%)	1.414	0.124-16.084	0.780

LGIN, low-grade intraepithelial neoplasia; HGIN, high-grade intraepithelial neoplasia.

### Risk Factors Affecting Curative Effect

Univariate logistic regression analysis was performed on the risk factors of potential non-en bloc resection and non-R0 resection. The results are shown in **Table 3**. We found that histology result of ectopic gastric mucosa was risk factor for non-en bloc resection (OR: 8.857, 95% CI: 1.378-56.915, P=0.022) and the lesion size ≥2 cm and histology result of HGIN were risk factors of non-R0 resection (OR: 12.606, 95% CI: 3.095-51.343, P < 0.001; OR: 9.111, 95% CI: 1.345-61.708, P=0.024). Further multivariate logistic regression analysis was performed on lesion size ≥2 cm and histology result of HGIN (**Table 4**.) The risk factors for non-R0 resection were lesion diameter ≥ 2cm (OR: 12.549, 95% CI: 2.950-53.379, P=0.001).

**Table 3:** Univariate logistic regression of efficacy indexes.

	Non En bloc Resection			Non R0 resection		
	OR	95%CI	P	OR	95%CI	P
Gender (Female)	0.784	0.139-4.439	0.784	0.327	0.068-1.575	0.163
Age (≥60)	0.382	0.043-3.371	0.387	0.724	0.183-2.869	0.646
Lesion Location (Bulb)	1.797	0.318-10.154	0.507	0.711	0.206-2.450	0.589
Lesion Location (Descending Portion)	0.489	0.055-4.330	0.521	2.255	0.646-7.873	0.202
Lesion Location (Junction Of The First And Second Portions)	0.939	0.105-8.422	0.955	0.448	0.055-3.674	0.454
Lesion Location (Horizontal Section)	–	–	1.000	–	–	1.000
Lesion Size (≥2cm)	–	–	0.996	12.606	3.095-51.343	<.001
Histology (LGIN)	1.270	0.223-7.233	0.788	0.535	0.110-2.594	0.437
Histology (HGIN)	6.350	0.596-67.692	0.126	9.111	1.345-61.708	0.024
Histology (Adenoma)	1.382	0.269-7.106	0.699	2.574	0.716-9.246	0.147
Histology (Adenocarcinoma)	–	–	1.000	–	–	1.000
Histology (Polyp)	0.324	0.037-2.854	0.310	0.152	0.019-1.224	0.077
Histology (Ectopic Gastric Mucosa)	8.857	1.378-56.915	0.022	3.778	0.682-20.915	0.128
Hypertension	–	–	0.998	0.898	0.182-4.418	0.894
Coronary Heart Disease	–	–	0.999	–	–	0.999
Diabetes	–	–	1.000	–	–	1.000
Cerebrovascular Diseases	–	–	0.999	–	–	0.999
Hepatitis B	–	–	0.999	–	–	0.999
Cigarette	1.426	0.250-8.141	0.689	1.679	0.461-6.112	0.432

LGIN, low-grade intraepithelial neoplasia; HGIN, high-grade intraepithelial neoplasia.

**Table 4:** Multivariate logistic regression of efficacy indexes.

Non R0 resection	OR	95%CI	P
Lesion Size (≥2cm)	12.549	2.950-53.379	0.001
Histology (HGIN)	8.967	0.913-88.057	0.060

HGIN, high-grade intraepithelial neoplasia.

## Discussion

SDELs are precancerous lesions of duodenal carcinoma (2). In the past, there was no effective measure to find early lesions, and it can only be treated by surgery after discovery, which has great trauma to patients, long recovery time, high cost and high risk (12). In recent years, with the development of endoscopy technology, upper gastrointestinal endoscopy has become a routine physical examination item, and has a strong ability to find gastroduodenal lesions (3). For early lesions, minimally invasive treatment can be carried out through endoscopy to achieve the curative effect (6). Compared with surgery, ER has less risk and better prognosis (6). However, the wall of the duodenum is thinner than that of the stomach, so the risk of complications of ER is higher when it carrying on in the duodenum. At present, lots of researches showed that ER is a safe and effective treatment of lesions in the duodenum (8, 9, 13, 14), in this article, we would focus on the therapeutic role of ER in SDELs. This research summarizes the data of patients who underwent ER of SDELs in our hospital from January 2010 to September 2021. The en bloc resection rate was 95.62% (131/137), R0 resection rate was 91.97% (126/137), the positive margin rate of postoperative pathological results was 3.65% (5/137), the postoperative bleeding rate was 2.19% (3/137), and no postoperative perforation to be found. The en-bloc resection rate of EMR in this study (112/114, 98.25%) is slightly higher than those reported in previous study (6, 15–20), this may be because the lesions size (average size was 1.11 ± 0.60 cm) managed by EMR in our study is relatively small. What’s more, our institution is a high-volume institution that actively carries out endoscopic treatment, which may obtain better curative effect (13, 21). These data show that ER is an effective and safe treatment for SDELs.

We also analyzed the potential risk factors for postoperative complications, but found no statistically significant indicators, which is inconsistent with some existing reports. Their results showed that lesions size, HGIN and tumor location distal to the ampulla were risk factors of postoperative bleeding (6, 22, 23). This may be due to the small number of patients with postoperative complications in the cases collected in this research, so a larger sample research may be needed. Previous studies have reported that the incidence of postoperative complications in ER is 1.5% - 15% (6, 9, 13, 23), and that in this study is 2.19% (3/137), which may be related to the less trauma of ER and the proficiency of the operator. It shows that ER is a relatively safe treatment for SDELs.

In addition, we also analyzed the risk factors for the efficacy indicators, i.e. en bloc resection and R0 resection. It was found that the histology result of ectopic gastric mucosa was the risk factor of non-en bloc resection and the lesion size ≥2 cm was the risk factor of non-R0 resection. Ectopic duodenogastric mucosa refers to the discovery of a large number of cells similar to gastric parietal cells and gastric main cells in the resected duodenal epithelium. The pathogenesis is not clear. At present, it is considered to be a congenital disease. Its relationship with ER needs more research to verify. Lesion size is an important factor for R0 resection. For larger lesions, in order to reduce the trauma to patients, reduce the difficulty of surgery, and ensure the treatment effect, non-en bloc resection is usually selected.

This research has some limitations. Firstly, this research is a single center retrospective research, and the extrapolation of conclusions should be cautious. Secondly, due to the incomplete preservation of clinical data in the past, although we have tried our best to fully collect the characteristics of patients, some characteristics still can not be emerged. And only 1 patient with adenocarcinoma was collected, so the conclusion may not be generalized to duodenal cancerous lesions. Thirdly, we did not compare the efficacy between different devices. And we did not compare ER with surgical treatment. However, this does not prevent us from summarizing and analyzing the limited characteristics. These data can be used as the basis for larger and more rigorous research in the future, or meta-analysis with published studies, which can more truly reflect the efficacy and safety of oral endoscopy in the treatment of SDELs.

## Conclusion

In conclusion, through the analysis of the collected data, we conclude that ER is a safe and effective method for the treatment of SDELs. The histology result of ectopic gastric mucosa was the risk factor of non-en bloc resection and the lesion size ≥2 cm was the risk factor of non-R0 resection.

## Data Availability Statement

The raw data supporting the conclusions of this article will be made available by the authors, without undue reservation.

## Ethics Statement

All patients have been fully informed and signed informed consent. This study was approved by the ethics review committee of the Second Xiangya Hospital of Central South University.

## Author Contributions

Research design: SF, DL, and YT. Administrative support and organization: YW, DL, and YT. Data collection and analysis: SF, JG, MZ, and YW. Manuscript drafting: SF, and YT. Modification and final approval: SF, JG, MZ, YW, DL, and YT. All authors contributed to the article and approved the submitted version

## Conflict of Interest

The authors declare that the research was conducted in the absence of any commercial or financial relationships that could be construed as a potential conflict of interest.

## Publisher’s Note

All claims expressed in this article are solely those of the authors and do not necessarily represent those of their affiliated organizations, or those of the publisher, the editors and the reviewers. Any product that may be evaluated in this article, or claim that may be made by its manufacturer, is not guaranteed or endorsed by the publisher.
